# “Voiceless Pain”—Assessment of Pain in Patients with Obstetric Brachial Plexus Injuries: A Retrospective, Single Center Analysis

**DOI:** 10.3390/jpm14101050

**Published:** 2024-10-10

**Authors:** Savas Tsolakidis, Bong-Sung Kim, Ziyad Alharbi, Rudolf Rosenauer, Robert Schmidhammer, Paul Supper

**Affiliations:** 1 Austrian Cluster of Tissue Regeneration and Ludwig Boltzmann Institute for Experimental and Clinical Traumatology, Research Centre for Traumatology of the Austrian Workers’ Compensation Board (AUVA), Donaueschingenstraße 13, 1200 Vienna, Austria; schmidhammer@millesicenter.com; 2 Department of Plastic Surgery and Hand Surgery, University Hospital Zurich, Raemistrasse 100, 8091 Zurich, Switzerland; bong-sung.kim@usz.ch; 3 Plastic Surgery and Burn Unit, Dr. Solaiman Fakeeh Hospital, Jeddah 23323, Saudi Arabia; zialharbi@fcms.edu.sa; 4 Clinical Sciences Department, Fakeeh College for Medical Sciences, Jeddah 23323, Saudi Arabia; 5 Trauma Hospital Lorenz Böhler of the Austrian Workers’ Compensation Board (AUVA), Donaueschingenstraße 13, 1200 Vienna, Austria; rudolf.rosenauer@auva.at; 6 University Clinic for Plastic, Reconstructive and Aesthetic Surgery, University Hospital Vienna, Währinger Gürtel 18-20, 1090 Vienna, Austria; paul.supper@meduniwien.ac.at

**Keywords:** obstetric brachial plexus injury, pain, pain coping strategies, brain plasticity, automutilation, microsurgical reconstruction

## Abstract

Background: Obstetric brachial plexus injuries (OBPIs) not only lead to severe and life changing sequelae regarding motor impairment but can also be responsible for multi-characteristic pain. In everyday routine, questions regarding pain of the developing child with an OBPI are often overseen and neglected. We aimed to elucidate this specific question and analyzed all patients with OBPI treated in our center to unmask initially non-observed pain and ultimately put pain in correlation to the surgical reconstructive treatment performed. Methods: This single center retrospective study analyzes patients with OBPI treated in our center over the past 20 years. Patients were surveyed by the adolescent pediatric pain tool assessment to evaluate pain over their entire life span by excluding potential postoperative pain episodes. Results: A total of 95 patients were initially contacted of which 78 returned the questionnaire (53.8% female, 46.2% male). In our patient cohort, the vast majority constituting 84.6 percent did not experience pain in the affected upper extremity over the years up to the date of their examination. Most of the patients describing pain had not been microsurgically treated for brachial plexus reconstruction in their neonate period. Merely, 33.3 percent of all OBPI experiencing pain had been microsurgically reconstructed at a median age of 7 months. Conclusions: Pain interrogation in patients with OBPI is often overseen during daily clinical routine. Adequate age-appropriate analgesic therapy regimens adapted to the individual are highly recommended. Timely microsurgical brachial plexus reconstruction may result in reduced lifetime pain experiences.

## 1. Introduction

Obstetric brachial plexus injuries (OBPI) constitute a significant neurological trauma witnessed at birth, impacting the newborn’s brachial plexus—a complex network of nerves responsible for the sensory and motor function of the upper extremity. OBPI can result in temporary or, in severe cases, permanent motor disability, often leading to significant functional impairments and psychosocial challenges, but in some cases also to undiscovered chronic pain. Moreover, due to muscular denervation, various muscular dysbalances due to skeleto-muscular maldevelopment could potentially lead to pain development on numerous levels. In Europe, the incidence of OBPI ranges from 3 to 4.6 per 1000 live births depending on cesarean sections and the protocols utilized to record affected children [1,2,3].

Pain, a multidimensional and subjective experience, is not merely a physical sensation but also carries emotional and cognitive facets. In children, pain perception is different than adults, due to developmental, physiological, and psychological factors. Research has shown that children’s pain perception is not just a scaled-down version of adult pain perception. Instead, children’s pain perception is influenced by the stage of their development, the pluripotent brain plasticity, and their life experiences of pain. All of that results in various consequences for their pain perception, pain behavior, and emotional response to pain [4]. The immature nervous system features a hypersensitivity to sensory stimuli with reduced thresholds to mechanical and thermal stimuli [4]. Experiences in this period of life mold the organization of various receptors, regulatory neurotransmitters, and ion channels of the central and peripheral nervous system [4,5]. Pain experiences in infancy also influence subsequent acquaintance with pain. Taddio et al. reported in 2002 of prolonged pain in terms of behavioral alterations consequently to repetitive heel lancing in neonates and possible reduced pain thresholds at later stages of their life [6].

The pluripotent brain plasticity, paired with the immaturity of the peripheral nervous system, may play a key role in the development of pain in neonates and children suffering from OBPI and their sequelae. This brain’s capacity to modify its connections and reorganize itself plays a crucial role in the context of OBPI and pain. More specifically, the concept of pluripotent brain plasticity in neonates signifies the potential to adapt and recover from injuries [7,8]. In children, this capacity is heightened, suggesting a strong therapeutic promise. There is evidence that in cases of upper root lesions, such as avulsion of C5 and C6, a shift of function in terms of functional compensation by the C7 root could be observed [7,8,9]. Posttraumatic sensory re-learning, based on brain plasticity after nerve injury, can be observed in children with a peak capacity below 5 to 10 years of age [10]. It has been hypothesized that this exceptional ability of the brain, paired with a delayed maturation of peripheral nerves in neonates, especially at the level of the Ranvier nodes, could be attributing factors to a lack of pain syndromes after trauma to the peripheral nerve system in the neonatal period [7,11,12]. However, this plasticity is a double-edged sword; while it enables adaptation and learning, it may also potentiate maladaptive responses, such as chronic pain following an injury [7,8]. Furthermore, their cognitive and emotional maturity is still in progress, which impacts their understanding, expression, and consequently the management of pain [7].

From a clinical perspective, recognizing pain in children with OBPI can be a daunting task, as they may not be able to communicate their experience verbally. In the context of OBPI, the perception and expression of pain can manifest through emotional distress, social withdrawal, sleeping and feeding difficulties, and subsequent challenges with daily activities, like dressing or playing. These daily hurdles can precipitate a vicious circle, reinforcing the pain experience and leading to further functional disability [13]. Adding to the problem, clinically recognizing pain in infants, young children, and adolescents is both challenging and significantly diverse. Faces pain scale, adolescent pediatric pain tool, pediatric pain scale, self-reported measures of pain, poker chip tool, and smiley scale are somehow objective multidimensional pain measurement tools of great importance but are dependent on the developmental maturity and thus reserved for children from 4 years of age and above [14]. Below this age, there are merely subjective parameters, like altered vital signs, grimacing, bulged brows, squeezed eyes, accentuated nasolabial folds, sleeping and feeding disorders, motor, and sensory, and vegetative marks, that prevail [15]. Supporting clinical evaluation between the ages of 0 and 4 can be provided by the suggested Infants and children’s pain scales of Büttner, including factors, like crying, facial expression, nasolabial fold, posture, arm, finger, leg position, sweating, and motor disquietude [16]. The mainstay of modern pain strategies in children and young adolescents is based on pharmacologic treatment, which includes stepwise treatment protocols standardized by the WHO and nonpharmacologic methods, like tactile stimulation, warmth, cuddling, and suckling, indispensably paired with the parents and supported by an adequate environment [17,18,19]. Chronic pain, especially neuropathic pain, represents a real challenge and is often underrecognized. Pediatric centers worldwide have followed a multidisciplinary approach when following their treatment protocols, consisting of psychological, pharmacological, physical, and behavioral pillars [17,20]. Pain coping strategies in general also markedly differ between children and adults. While adults can articulate their pain, use cognitive behavioral techniques, and seek medical and social support, neonates and children rely on distraction, play, parental reassurance, and regression [4]. In some cases, children can also mirror their pain experience and feelings through drawings or paintings [21]. Considering the fact that early life experiences can configure pain processing and sensitivity throughout life, it is of uttermost importance to recognize pain early enough and consequently establish age-appropriate multidisciplinary treatment plans that encompass pharmacological, physical, and psychological therapies to optimally manage OBPI-related pain. Completively, alternative treatment modalities, e.g., hypnosis, diversionary tactics, and acupuncture, should not be overlooked, completing the armamentarium of pain control [4,22]. Overall, the assessment and treatment of pediatric pain requires a sensitive, patient-centered approach, factoring in the child’s developmental stage, cognitive abilities, and emotional states. In addition, children may experience pain as a feeling of discomfort or as an altered sensation [23]. Moreover, potential barriers include the lack of standardized pain measures in every-day clinical practice, communication difficulties, and misconceptions about children’s ability to experience an express pain. This retrospective study aims to evaluate pain in neonates, children, and young adolescents with OBPI, which had been treated in a single center, while shedding light on the role of brain plasticity and daily life compensatory strategies. Above all, the study tries to develop an appropriate awareness paired with feasible treatment strategies for these severely injured young patients.

## 2. Materials and Methods

This study included all patients with obstetric brachial plexus lesions who completed a pain questionnaire between 2003 and 2023 at the Millesi Center, a center of excellence for peripheral nerve surgery and brachial plexus surgery located in Vienna, Austria. The primary research questions addressed the lifetime prevalence of pain in patients with OBPI, and the secondary research question dealt with the influence of primary vs. secondary therapy on the prevalence of pain. After screening data collection of age, biological sex, classification of OBPI, time of treatment, and treatment method was conducted pseudonymized (consecutive numbering of all patients). Surgical treatment was classified as microsurgical reconstruction of the brachial plexus, including neurolysis, nerve grafting, and nerve fiber transfers, as well as secondary procedures on a musculoskeletal level.

A pain questionnaire, based on the adolescent pediatric pain tool assessment, including age of first clinical examination and/or treatment in our center and utilizing children’s appropriate pain descriptors for the affected as well as the unaffected body side, had been evaluated. This questionnaire was provided during treatment to the patients or their parents. During the follow-up examination, which was conducted by one reconstructive surgeon, the patients above the age of 5 years were asked if pain sensations were present. In case of a positive answer, a more detailed questionnaire had been elaborated, decoding pain details, such as pain character, incidence, and location. In cases where toddlers and preschoolers had been enrolled in the study, both parents were investigated and asked specifically, regarding variances in sleep, feeding, and behavior. Shifting of pain, meaning pain variations in frequency and body region over time, were incorporated as well. To decipher existing discomfort, which had not been appraised as pain in the first place because of their existence since birth, we also asked the patients if the affected area felt somehow strange/alien from the healthy one. The questionnaires are pseudonymized and transferred to a password-protected Excel spreadsheet within the clinic. Only persons within the clinic have access to the questionnaires and the original Excel file, which are protected by a double password query. Sample size calculations were based on the data of Ho et al., 2015 [23], recording a lifetime pain prevalence of 25%, resulting in >21 individuals per group. (Univie sample size calculator, version 1.061; https://homepage.univie.ac.at/robin.ristl/samplesize.php? Accessed on: 6 February 2024). Statistical calculation was performed using IBM SPSS 24 (Armonk, NY, USA). Normal distribution was tested using the Kolmogorov–Smirnov test. A Mann–Whitney U test was used for not normally distributed variables. Differences are considered significant from a probability of error of less than 5% (*p* < 0.05).

## 3. Results

Between 2003 and 2023, a total of 95 children with OBPI treated at the center were eligible for data collection. In 82.1%, a total of 78, the pain questionnaire and documented follow-up were available for evaluation. Of these, 27 patients were infants (age 0–1), seven were at a toddler age (age > 1–2), 15 were preschoolers (3–5), 15 were elementary children (>5–10), four preteens (>10–12), seven were teenagers (>12–19), and three young adolescents when treated surgically for the first time. In general, 27 patients, representing 36.4 percent of all patients, underwent microsurgical reconstruction on the level of the brachial plexus at a median age of 6.9 ± 2.8 months. The age of the patients ranged at the time of their clinical evaluation for this pain study from 4 months to 26 years, with a median age of 15.7 ± 9.1 years. 53.8 percent were female, and 46.2 percent were male. In total, 19 patients (24.3%) sustained a complete OBPI, 28 (35.9%) an Erb’s palsy, and 25 (32%) a lesion of C5, 6, and 7. No patient with Klumpke palsy was included. However, there was one (1.3%) patient with an isolated lesion of the root C5, one (1.3%) with an isolated lesion of C6 and C8, and four patients (5.1%) with lesion in the root C5, 6, 7, or C8. Of our total number of patients, 78 (82.1%) responded to our questionnaire. In general, 12 patients (*n* = 15.3%) reported pain sensations in the affected upper limb (Table 1).

When analyzing pain, we asked for lifetime prevalence of pain and not prevalence of pain, at the time of completing the questionnaire. Pain was described as cramp-like by six patients (7.8%), as neuropathic by four patients (5.1%), and as stabbing by two patients (2.6%) (Table 2).

No statistically significant accumulation of a pain characteristic was found (*p* > 0.05). Most mentioned parts of the body affected with pain were in declining order, the ventral and dorsal parts of the shoulder, secondly the shoulder region, followed by the back of the hand, and in one case the whole arm (Table 3).

Pain has been described as episodic in nature by all patients. None described pain in the unaffected upper extremity. One patient who suffered from the sequelae of an Erb’s palsy described a strategy to cope with the cramp episodes in his arm by wrapping his entire arm with a rope for minutes, sometimes hours. All other patients did not integrate specific coping strategies to manage their pain episodes, except of moving the affected extremity in any direction and in any way, aiming for pain relief. In two patients, pain worsened during fever periods. When breaking down these patient cases reporting pain in accordance with their level of nerve damage, four patients (33.3%) had sustained an extended upper root lesion in C5, 6, and 7; five patients (41.7%) had been diagnosed with an upper root lesion; and three patients (25%) presented with a lesion of all roots. No statistically significant increased rate of pain was found when a specific nerve root was involved (*p* > 0.05). Further deciphering the number of children with OBPI and episodic pain, in conjunction with performed microsurgical reconstructive methods revealed that merely four children out of 12, representing 33.3% underwent a microsurgical reconstruction on the level of the brachial plexus at the median age of 7 months, whereas the remaining majority with OBPI, representing 66.7% of children and young adolescents (*n* = 8) reporting of pain did not undergo a microsurgical reconstruction of the brachial plexus as their first line treatment, but were functionally reconstructed by secondary procedures, essentially muscle/tendon transfers as their exclusive surgical treatment at a later life-age, a median age of 27 months. Thereby, reduction of occurrence of pain was reduced statistically not significant (*p* > 0.05) (Table 4).

## 4. Discussion

Our study reveals that patients which underwent a reconstruction of the brachial plexus upon OBPI reported a reduced rate of pain compared to patients which only underwent secondary procedures to improve the function of the affected limb, although this difference was statistically not significant. Several previous studies have undermined the fact that surgical reconstruction of the injured brachial plexus not only bears the prospect of a functional re-integrity of the affected upper extremity, but also reduces pain or the risk of pain development [23,24]. Independent of the surgical methods used, only four out of 12 patients with pain symptoms over time had been reconstructed microsurgically on the brachial plexus level in our cohort. There is evidence, that microsurgical nerve reconstruction can lead to significant reduction of pain in brachial plexus patients [23], when compared to studies where patients with OBPI never experienced nerve reconstruction [24,25]. Adding to this, also non-surgical rehabilitative methods, e.g., Intention Controlled Myofeedback (IMF), utilized as an adjunct in physical therapy and rehabilitation after OBPI lesions are capable of empowering regenerative improvement on a motor and sensory level by reorganization on a cortical and peripheral level. By providing a cortical re-modeling non-surgical methods can lead to significant reduction of pain [26,27].

Independent of the spinal nerve roots affected, microsurgical nerve grafting alone or in combination with nerve fiber transfers leads to a promising approach in terms of re-establishing the cortical representation in the central nervous system and thus reducing possible pain. International brachial plexus and peripheral nerve centers aim to reconstruct the brachial plexus and its motor and sensory function as anatomically genuine as possible with all available surgical options in each individual case. Failure of restoration could result not only in poor functional outcome but also neuropathic pain. If a central cortical–periphery connection can be re-established, brain mapping can be saved and thus pain, which in many cases represents loss of this connection reduced or even nullified. Similar to our findings, Partridge et al. reported that patients without surgical reconstruction of upper root lesions presented with pain, with one third of them describing neuropathic pain with the tendency to worsen over the years [25]. In accordance, Anand and Birch reported lack of pain and/or neuropathic pain in the majority of patients with severe OBPI, in which 83 percent had been microsurgically reconstructed on the brachial plexus level under the age of 12 months [7]. On the contrary, Ho et al. demonstrated that lifetime prevalence of pain was present in 66% of their patients, of which all had undergone microsurgical reconstructions under the age of 12 months [23]. Pain perception in children is challenging and depends on various factors, e.g., ethical background, gender, familial origin, coping strategies, constitution during day and night, and possible view of their pain as part of their everyday life and existence [23,28,29]. Moreover, it is of uttermost importance to consider some indisputable preconditions when dealing with these patients in daily routine. Key factors include utilizing an age-appropriate language and age-dependent descriptive words, include the parents, and the seting up of an appropriate environment for a clinical examination and interrogation. A challenging question remains, when dealing with younger children with OBPI, who are presenting for a pain interrogation at a pre-verbalizing (competence) developmental level. Care must be taken to meticulously observe the affected child, also regarding automutilation, meaning loss of parts of the extremity due to excessive mouthing and/or biting. This phenomenon may represent the possible presence of pain or dysesthesia, as suggested by Al-Qattan and McCann et al. [30,31]. Interestingly, McCann et al. revealed in their study with 280 patients suffering from the sequelae of OBPI that many more patients were affected by automutilation in the microsurgically reconstructed group than in the non-surgical group [31].

Obstetric brachial plexus injury patients also reveal various dysharmonic and pathologic muscle innervation, leading to abnormal biomechanical forces, which can cause additional stress on joints and muscles [25]. Adequate to this, Ruoff et al. could demonstrate in 2012 that specific muscles, such as the elbow flexors and extensor muscles, are reduced in volume after birth in OBPI cases, adding to the biomechanical dysbalance [32]. Complementary, Bellew et al. centered arising developmental and behavioral problems around an additional mid-line cortical orientation in OBPI [33]. The importance of evaluating pain in these patients is also reflected in later reported challenges, such as limitations in everyday activities, e.g., cooking, bathing, and dressing themselves, as reported by Partridge et al. in 2004 [25]. Accumulative motor problems, paired with undiscovered and untreated pain, can result in further restrictions and psychological stress, leading to a vicious circle [25]. Other studies indicate that adolescents with obstetric brachial plexus palsies, of which 33 percent had been microsurgically reconstructed on the brachial plexus level, and had interests, activities, and a social life, just like uninjured teenagers in the comparison group, although differences had been described regarding a lower self-esteem in sports and motor activities due to their limitations [34]. Aside from focusing on the reconstruction of the anatomy, regarding the lesioned brachial plexus, the question of whether pain is present or not should be raised in everyday routine when dealing with those patients. Besides utilizing charts, e.g., the faces pain scale, the children’s pain scale, or related ones [14,15,16], nowadays new perspectives with the support of computer-assisted programs, smart phones, and artificial intelligence may have the potential to broaden the clinical horizon and add significant value to pain assessment [35,36]. Limitations of our study are the retrospective design of the study with a long timespan between initial treatment and study assessment. Furthermore, data on pain perception were not analyzed regarding ethnic background and/or decent. Previous studies analyzing general pain in ethnically diverse patients substantiated differences in pain perception and assessment [37]. Nonetheless, the long-term follow-up to adulthood and the reduced rate of reported pain in children where a surgical reconstruction of the brachial plexus had been performed may support the significance and importance of a timely microsurgical reconstruction, not only to restore function but also to reduce or avoid pain.

## 5. Conclusions

Pain is a burden that should not be overseen when dealing clinically with children and young adolescents suffering from the sequelae of obstetric brachial plexus lesions. In our patient cohort, which includes observation and clinical examination over a time span of 20 years, pain was mainly present in children without microsurgical reconstruction of the initial brachial plexus lesion, at best within 12 months after birth. Pain was described as cramps, electric sensations, stinging, and hypesthesia in the majority of patients.

Further clinical studies are needed to enlighten pain in OBPI children and young adolescents with the aim to establish standardized questionnaires and protocols, which should be a fixed part of any clinical examination and interrogation. To be able to offer a holistic therapeutic approach to this vulnerable patient collective, not only function should be in our focus, but pain as well. Recognizing and treating pain in multifaceted ways and on various levels, e.g., IMF, hypnosis, diversionary tactics, parent-guided non-medical therapies, massage, acupuncture, etc., should find its way into our daily clinical setting and positively replenish the analgetic armamentarium [4].

## Figures and Tables

**Table 1:** Numeric and percentage overview of the examined patient cohort with a comparison of pain and no pain patients. It was found that more patients without microsurgical nerve reconstructions experienced pain than those who underwent microsurgery. Timely microsurgical reconstruction may reduce the risk of lifetime pain, although not statistically significant.

	Number of Pain Patients *n* = 12	Number of No Pain Patients *n* = 66	Ratio of Pain/No Pain Patients in Percentage (%) in Relation to the Total Number of Patients (*n* = 78)	*p* Value
Microsurgery	4	25	5.1/32	>0.05
No Microsurgery = Secondary Procedures only	8	41	10.2/52.6	>0.05

**Table 2:** Pain descriptors, frequency of pain, and potential maneuvers for pain control. The overview represents a complete questionnaire, which had been utilized for all patients during or after a thorough clinical examination. This overview includes merely the pain patients cohort. Pain was described as cramp-like by six patients (7.8%), as neuropathic by four patients (5.1%), and as stabbing by two patients (2.6%).

Pain Descriptive Words	Number of Patients Affected Total: *n* = 12	Frequency (Daily, Weekly, Monthly, Yearly)	Maneuver for Pain Relieve Yes/No
Like a Sting	0	0	N
Tight/Constriction Feeling	0	0	N
Like a Pinprick	0	0	N
Like a Cramp	6	Weekly	N
Like a Stab	2	Monthly (*n* = 1) Yearly (*n* = 1)	N
Numbness	0	0	N
Feeling of Pressure	0	0	N
Burning	0	0	N
Itching	0	0	N
Hot	0	0	N
Electric	4	Monthly	Y (*n* = 1)
Uncontrollable	0	0	N
Unconfortable	0	0	N

**Table 3:** Overview of the pain location in accordance with the obstetric brachial plexus root lesion involved (the number of patients is put in brackets). No statistically significant accumulation of a pain characteristic was found (*p* > 0.05). Most mentioned parts of the body affected with pain were, in declining order, the ventral and dorsal parts of the shoulder; secondly, the shoulder region; followed by the back of the hand; and, in one case, the whole arm.

Location of Pain Episodes	Complete OBPI (C5,6,7,Th1) *n* = 3	Extended Upper Root Lesion (C5,6,7) *n* = 4	Upper Root Lesion (C5,6) *n* = 5
Shoulder (ventral)	x (*n* = 1)	x (*n* = 1)	x (*n* = 1)
Shoulder (dorsal)		x (*n* = 2)	x (*n* = 2)
Upper Arm (ventral)	x (*n* = 1)	x (*n* = 2)	x (*n* = 2)
Upper Arm (dorsal)		x (*n* = 1)	x (*n* = 1)
Lower Arm (ventral)	x (*n* = 1)		
Lower Arm (dorsal)	x (*n* = 1)	x (*n* = 1)	
Arm complete	x (*n* = 2)	x (*n* = 1)	x (*n* = 1)
Hand	x (*n* = 1)		x (*n* = 1)

**Table 4:** Demographic distribution of pain patients in accordance with the performed surgical strategy. Pain was less present in patients who underwent a microsurgical brachial plexus reconstruction, but this finding was statistically not significant (*p* > 0.05).

	Number of Pain Patients *n* = 12	Microsurgery	Secondary Procedures	*p* Value
Complete OBPI (C5,6,7,8,Th1)	3	3	0	>0.05
Extended Upper Root Lesion (C5,6,7)	4	0	8	>0.05
Upper Root Lesion (C5,6)	5	1	0	>0.05

## Data Availability

The data presented in this study are available upon request from the corresponding author (specify the reason for the restriction).
